# Coding-Complete Genome Sequence of a Lumpy Skin Disease Virus Isolated during the 2021 Thailand Outbreak

**DOI:** 10.1128/mra.00375-22

**Published:** 2022-07-13

**Authors:** Weena Paungpin, Ladawan Sariya, Somjit Chaiwattanarungruengpaisan, Metawee Thongdee, Bunlue Kornmatitsuk, Akarapong Jitwongwai, Sarawut Taksinoros, Kripitch Sutummaporn, Sookruetai Boonmasawai, Chowalit Nakthong

**Affiliations:** a Monitoring and Surveillance Center for Zoonotic Diseases in Wildlife and Exotic Animals, Faculty of Veterinary Science, Mahidol University, Salaya, Nakhon Pathom, Thailand; b Department of Clinical Sciences and Public Health, Faculty of Veterinary Science, Mahidol University, Salaya, Nakhon Pathom, Thailand; c Department of Pre-clinic and Applied Animal Science, Faculty of Veterinary Science, Mahidol University, Salaya, Nakhon Pathom, Thailand; KU Leuven

## Abstract

Lumpy skin disease (LSD) is an economically devastating and transboundary disease in cattle. Here, we report the coding-complete genome sequence of the LSDV72/PrachuapKhiriKhan/Thailand/2021 strain, which was isolated from an affected cow during the first LSD outbreak in Thailand in 2021. The sequence will be beneficial for future genomic studies of the virus.

## ANNOUNCEMENT

Lumpy skin disease virus (LSDV), which is a causative agent of lumpy skin disease (LSD), is a member of the genus *Capripoxvirus* within the family *Poxviridae*. LSDV is a double-stranded DNA virus and has an ~151-kbp genome. Historically, LSD was endemic to the African continent; however, the disease has now spread to the Middle East, Europe, and Asia (1). In 2019, LSD was first reported in Asia and the Pacific region (Bangladesh, China, and India) and then spread to affect Taiwan, Myanmar, Vietnam, Thailand, and Hong Kong territory (2–7). Here, we report the coding-complete genome sequence of an LSDV strain that was isolated from an LSDV-infected cow during the first LSDV outbreak in Thailand in 2021.

The LSDV72/PrachuapKhiriKhan/Thailand/2021 strain was isolated from a skin nodule of an affected cow in Prachuap Khiri Khan province, Thailand. The sample was isolated on a Madin-Darby bovine kidney cell line (NBL-1 [ATCC-CCL-22]) following the procedure described by Salnikov et al. (8) with some modifications. DNA was extracted from the culture supernatant using a QIAamp DNA blood midi kit (Qiagen, Hilden, Germany). Next-generation sequencing libraries were constructed following the manufacturer’s protocol (NEBNext Ultra DNA library preparation kit for Illumina; New England Biolabs, MA, USA). The concentration of the generated libraries was 15.39 nM. Sequencing was performed on a HiSeq platform (Illumina, CA, USA) using a 2 × 150-bp paired-end configuration. Quality control was performed for the raw sequences, and the adapter and primer sequences were trimmed via fastp v0.20.1 (https://github.com/OpenGene/fastp). The sequence quality assessment was reported using FastQC v0.11.5 and MultiQC v1.7 tools. Reads mapping to the host genome (Bos taurus [GenBank accession number NC_037328.1]) using Bowtie2 v2.3.4.1 were removed from the cleaned sequences, and 1,176,127 reads remained. These remaining reads were *de novo* assembled using SPAdes v3.11.1 with a minimum contig size of 500 bp. The assembly resulted in the near-complete genome (150,602 bp, with a GC content of 25.89%) of isolate LSDV72/PrachuapKhiriKhan/Thailand/2021. The genome coverage was 2,343× (the number of reads in this contig was multiplied by the read length in paired-end mode and subsequently divided by the length of the reference genome). The genome contains a central coding region flanked by two inverted terminal repeats of at least 2,358 bp and all expected LSDV open reading frames. The identity analysis was performed using the nucleotide BLAST program (https://blast.ncbi.nlm.nih.gov/Blast.cgi) with default parameters. The results indicated that the LSDV72/PrachuapKhiriKhan/Thailand/2021 isolate shares 100% and 99.98% nucleotide identity with a contemporary recombinant LSDV strain from China (China/GD01/2020 [GenBank accession number MW355944.1]) and four isolates from Vietnam (GenBank accession numbers MZ577073.1 to MZ577076.1), respectively. Annotation and amino acid gene prediction were performed using GATU software (https://4virology.net/virology-ca-tools/gatu). Amino acid changes were identified in the coding sequence alignments of the China/GD01/2020 and Bang-Thanh/VNM/20 (GenBank accession number MZ577076.1) strains (Table 1). These findings highlight the importance of obtaining the coding-complete genome sequence of an LSDV strain from the 2021 outbreak in Thailand to support future genomic studies of the virus.

**TABLE 1:** Amino acid changes within the genome of the LSDV72/PrachuapKhiriKhan/Thailand/2021 isolate, compared with closely related isolates

Gene	Predicted function	Amino acid changes^*a*^
Compared with China/GD01/2020		
LSDV001	Hypothetical protein	E42V
LSDV154	Putative endoplasmic reticulum-localized apoptosis regulator	R171S
LSDV155	Hypothetical protein	S80T, X128N, K132stop codon
Compared with Bang-Thanh/VNM/20		
LSDV023	Hypothetical protein	P60L
LSDV144	Kelch-like protein	L547F, stop codon548V (amino acids VKT inserted at positions 548–550)

a The amino acid changes are indicated using the format XnY, where X indicates the amino acid in China/GD01/2020 (GenBank accession number MW355944.1) or Bang-Thanh/VNM/20 (GenBank accession number MZ577076.1), n is the amino acid number of the respective gene, and Y is the amino acid in LSDV72/PrachuapKhiriKhan/Thailand/2021. X, unknown amino acid (degenerate base K present in the gene sequence, resulting in an unknown amino acid).

### Data availability.

The coding-complete genome sequence of the LSDV72/PrachuapKhiriKhan/Thailand/2021 isolate has been deposited in GenBank under the accession number ON152411. The raw data have been deposited in the SRA database under the BioProject accession number PRJNA823388.
